# Measurement, Classification and Evaluation of Sleep Disturbance in Psoriasis: A Systematic Review

**DOI:** 10.1371/journal.pone.0157843

**Published:** 2016-06-21

**Authors:** Alasdair L. Henry, Simon D. Kyle, Sahil Bhandari, Anna Chisholm, Christopher E. M. Griffiths, Christine Bundy

**Affiliations:** 1 Centre for Dermatology Research, Manchester Academic Health Science Centre, University of Manchester, Manchester, United Kingdom; 2 Manchester Centre for Health Psychology, University of Manchester, Manchester, United Kingdom; 3 Sleep and Circadian Neuroscience Institute, Nuffield Department of Clinical Neurosciences, University of Oxford, Oxford, United Kingdom; 4 Manchester Medical School, University of Manchester, Manchester, United Kingdom; 5 Salford Royal NHS Foundation Trust, Salford, United Kingdom; University of California, San Francisco, UNITED STATES

## Abstract

**Background:**

Psoriasis is a long-term immune-mediated inflammatory disorder mainly, but not only, affecting skin, and is associated with significant medical and psychological morbidity. Evidence suggests that sleep is disrupted in psoriasis, however high quality empirical evidence is lacking. Given the importance of sleep for health, characterisation of sleep disruption in psoriasis is an important goal. We therefore conducted a systematic review of the sleep-psoriasis literature.

**Methods:**

Searches were conducted in Pubmed, SCOPUS and Web of Science from inception to May 2016. Studies were compared against inclusion/exclusion criteria and underwent a quality evaluation. Given the heterogeneity of studies, we conducted a narrative synthesis of the findings.

**Results:**

Searches revealed 32 studies which met our predetermined inclusion/exclusion criteria. Whilst 93.7% of studies reported sleep disruption in this population, ranging from 0.05% to 85.4%, many had important methodological shortcomings. Over half of all quantitative studies (54.8%; 17/31) relied on non-validated measures, contributing to heterogeneity in study findings. In those that employed valid measures, assessing sleep was often not the primary objective. We frequently found the absence of adequate sample size calculations and poor statistical reporting.

**Conclusion:**

This review showed that in psoriasis, reported sleep rates of sleep disturbance varied substantially. Most studies lacked a hypothesis driven research question and/or failed to use validated measures of sleep. We were unable to draw firm conclusions about the precise prevalence and nature of sleep disturbance within the psoriasis population. We offer suggestions to help advance understanding of sleep disturbance in psoriasis.

## Introduction

Psoriasis is a long-term, immune mediated inflammatory skin disorder, characterised by scaly plaques on knees, elbows and scalp, but any skin surface may be affected [1]. Population-based cohort studies indicate that psoriasis is associated with other systemic inflammatory conditions, including: psoriatic arthritis (PsA) [2]; inflammatory bowel disease [3]; cardiovascular disease (CVD) [4]; and diabetes [5].

Contemporary evidence suggests that sleep is important for daytime functioning and health, [6–8] subserving optimal physiological [9] and psychological functioning [10–13]. Moreover, disturbed sleep may causally drive disease processes. For example, persistent sleep disturbance is a risk factor for the future development of diabetes [14], CVD [15, 16], hypertension [17,18], and depression [19–22]. Several studies [23–38], including four reviews, have examined sleep domains in psoriasis populations. One review evaluated factors linked to sleep disturbance in psoriasis, concluding that mood, obstructive sleep apnoea (OSA), itch and pain as possible sources of sleep disturbance [29]. Another review provided an overview of the sleep and dermatology literature with a focus on the role of itch, suggesting skin temperature, circadian rhythm and psychological factors, such as depression, influenced itch, which in turn disrupted sleep [30]. Similarly, a further review assessed the associations between sleep disorders and the broad category of skin disorders [31]. More recently, Gupta et al. conducted a systematic review of psoriasis and sleep disorders and found an increased prevalence of OSA and restless leg but inconclusive evidence of increased insomnia risk [32]. Whilst these reviews provide some insight into the nature and predictors of sleep in psoriasis the majority are narrative and none critically examine the measures used to assess sleep nor do they assess the quality of current research. Additionally the most recent review [32] only included papers where a sleep disorder diagnosis was made, thus excluding studies where sleep was assessed without a diagnosis, potentially missing research where sleep was assessed but no diagnosis was made. Accordingly, a more rigorous and nuanced appraisal of the sleep-psoriasis literature is required. We present a systematic review focusing on (1) the evidence for sleep disturbance in psoriasis and (2) the quality of measures and methodology employed to assess sleep in patients with psoriasis. We conclude by outlining a research agenda aimed at advancing understanding of sleep disturbance in the context of psoriasis.

## Method

### Search criteria

PubMed, SCOPUS and Web of Science databases were searched from inception to May 2016. We used the PICOS framework (Population, Interventions, Comparators/Control, Outcomes, Study design) [33] however the intervention category could not be applied due to poor reporting and/or too much variation in study design. In order to increase the sensitivity of the search, search terms were kept broad and were combined using ‘AND’ plus wildcard operators (*) and MeSH terms (Table 1). Studies were assessed against inclusion/exclusion criteria determined by an expert group of reviewers (Table 2). Reference lists of eligible studies were forward and backward searched for completeness. Reviews were included to identify additional studies that were not identified via database searches.

**Table 1:** Search terms used across databases.

1. Sleep /OR sleep disruption /OR sleep disturbance /OR sleep fragmentation
2. Psoriasis/ OR chronic plaque psoriasis/ OR psori*
3. Circadian*/ OR circadian rhythm
4. 1 OR 3
5. 4 AND 2

**Table 2:** Inclusion criteria for studies.

Inclusion Criteria	Exclusion Criteria
1. Partial or whole psoriasis sample (including any subtype of psoriasis or psoriatic arthritis)	1. Not peer reviewed
2. Sleep was measured either directly using a valid measure of sleep, unvalid measure of sleep or by-proxy via another measure	2. Animal studies
3. Any study design	
4. Any age group	
5. Published in English	
6. Accessible in full text	

### Data extraction

Data extracted from studies included: authors, title, year, study aim(s), journal, study design, type of psoriasis, sample size, measure of sleep used and findings relating to sleep (Tables 3–5).

**Table 3:** Information (i.e. aim, study design, primary focus of the study, measure of sleep used, sample, pertinent results relating to sleep) and quality scores for each study using a validated measure of sleep included in the review.

Type of measure of sleep	Study	Aim	Study Design	Primary focus on sleep	Measure of Sleep	Participants	Results pertaining to sleep	Quality Score	Quality Percentage	Quality Decision
Validated subjective	Gezer et al., 2014	Determine the effects of PsA on sleep quality and associations between sleep, QoL and psychological state in PsA	Case-control study	Yes	PSQI	41 patients with psoriatic arthritis and 38 healthy controls	Subjective SQ, SOL, SD, SE, sleep disturbance, daytime dysfunction and total PSQI scores were all significantly higher (worse) in PsA patients (9.70 ± 3.90) compared to controls (4.05 ± 1.85) (P < 0.05)	26/34	76.5	fair
Validated subjective	Stinco et al., 2013	Investigate the influence of Psoriasis on sleep	Case-control study	Yes	PSQI	202 patients with psoriasis and 202 healthy controls	No significant difference in PSQI score between psoriasis patients (5.56 ± 3.93) and controls (5.13 ± 4.16) (P > 0.05).	25/34	73.5	fair
Validated subjective	Balta et al., 2015	To investigate sleep quality, general psychiatric symptoms and coping strategies in psoriasis	Case-control study	Yes	PSQI	37 patients with psoriasis and 42 control subjects	Significant differences in subjective sleep quality between psoriasis patients (1.48 ± 0.17) and controls (1.02 ± 0.13) (P < 0.05) and in habitual sleep efficiency between psoriasis patients (0.79 ± 0.19) and controls (0.32 ± 0.14) (P < 0.05).	24/34	73.5	fair
Validated subjective	Shutty et al., 2013	Measure prevalence of sleep disturbance in psoriasis	Case control study	Yes	PSQI, ISI, ESS	35 patients with psoriasis and 44 controls	PSQI scores higher in psoriasis patients (8.8 ± 4.4) than controls (6.3 ± 4.4) (P < 0.05). ISI scores significantly higher for psoriasis patients (11.0 ± 7.0) than controls (6.3 ± 6.0). (P < 0.05). ESS scores were not significantly different.	25/34	73.5	fair
Validated subjective	Ljosaa et al., 2012	Investigate the association between skin pain/discomfort and HRQoL and to explore whether sleep disturbance is a mediator of this relationship	Cross-sectional study	No	GSDS	139 patients with psoriasis	Mean GSDS score = 52.8, with the highest levels being reported in the pain group (66.6). Sleep emerged as a partial mediator for the association between skin pain and HRQoL	28/30	93.3	good
Validated subjective	Mrowietz et al., 2014	Characterize the extent of pruritus in moderate to severe psoriasis, and its association with QoL, pre and post exposure to varied doses of etanercept	RCT—post hoc analysis	No	MOS-SS	270 patients with plaque psoriasis	Levels of pruritus were significantly associated with MOS-SS scores (p < 0.05) (no pruritus = 22.17; mild-moderate = 29.60; severe = 37.52)	31/36	86.1	good
Validated subjective	Strober et al., 2012	Describe baseline sleep disturbance in psoriasis, factors associated with sleep disturbance, assess the impact of adalimumab on psoriasis and the correlation between sleep outcomes following treatment	Uncontrolled clinical trial	yes (but an embedded study)	MOS-SS	152 patients with psoriasis	Poor sleep was associated with lower DLQI scores at baseline (P < 0.05). Depression and PsA were significantly associated with daytime somnolence and sleep adequacy respectively. Following treatment, sleep improved at a MCID (30% improvement for sleep disturbance, 32.2% for daytime somnolence and 37% for perceived sleep adequacy)	19/30	63.3	fair
Validated sleep questionnaire	Zachariae et al., 2008	Validate a sensory and affective approach to understanding pruritus and explore associations between pruritus, psychological symptoms and perceived impairment of pruritus QoL. Evaluate the role of sleep disturbance as a mediator between pruritus, psychological symptoms and QoL.	Cross-sectional study	No	SQQ (3 items from PSQI)	40 patients with psoriasis	Impaired sleep quality partially mediated the association between itch severity and psychological symptoms.	24/30	80	good
Validated subjective	Thaci et al., 2014	Evaluate the safety and efficacy of etanercept alongside topical treatments on psoriasis	RCT	No	MOS-SS	270 patients with psoriasis	Mean baseline scores indicated sleep impairment. At baseline, somnolence and symptoms of OSA were 37% and 39% worse than the pop. norm. At follow up these improved to within 10% of the pop. norm. Sleep adequacy was 10% worse, improving to within 1% at follow-up. Sleep disturbance improved from 43% worse to 9%.	28/36	77.8	fair
Validated objective	Buslau & Bentomane., 1999	Assess the prevalence of OSA in psoriasis	Case control study	Yes	Polysomnography	25 patients with psoriasis and 19 matched controls with chronic bronchitis	OSA occurred at greater rates in psoriasis patients compared to those with chronic bronchitis (AHI: 14.4 vs 8.8)	20/34	58.8	poor
Validated objective	Maari et al., 2014	Assess the efficacy of adalimumab on sleeping parameters in patients with psoriasis and OSA	RCT	Yes	Polysomnography	20 patients with psoriasis and OSA	No significant difference between adalimumab and placebo groups from baseline to follow-up on AHI, SOL, SE, TWT, FOSQ, ESS or daytime SOL (p > 0.05).	30/40	75	fair
Validated objective	Papadavid et al., 2013	Determine the association between psoriasis and OSA taking into account demographic and metabolic parameters	Cross-sectional study	Yes	Polysomnography	35 patients with psoriasis	No correlation between OSA and psoriasis. When adjusting for psoriasis, age and gender, there was a significant association between OSA, BMI and hypertension (P < 0.05).	28/34	82.4	good
Validated objective	Karaca et al., 2013	Determine the frequency of OSA in psoriasis and its relationship with DLQI and psoriasis severity	Cross-sectional study	Yes	Polysomnography	33 patients with psoriasis	Frequency of OSA in psoriasis patients was found to be higher than within the normal population (54.5% vs 2–4%)	26/34	76.5	fair
Validated objective	Savin et al., 1975	Assess scratching during sleep	Cross-sectional study	Yes	Polysomnography	15 patients with varied dermatological disorders (5 with, 5 with dermatitis herpetiformis, 3 with lichen planus, 1 with urticaria and 1 with psoriasis)	Scratching was found to be most prevalent in stage 1 for all participants, with its frequency decreasing through stages 2,3 and 4.	16/30	53.3	poor

AHI—Apnoea Hypopnoea Index, BMI—Body Mass Index, BPI (30)–Brief Pain Inventory, ESS–Epworth Sleepiness Scale, FOSQ—Functional Outcomes of Sleep Questionnaire, GSDS–General Sleep Disturbance Scale, HRQoL/QoL—Health related quality of life/Quality of Life, ISI–Insomnia Severity Index, MCID–Minimal Clinically Important Difference, MOS-SS–Medical Outcomes Study Sleep Scale, OSA–Obstructive Sleep Apnoea, PSA–Psoriatic Arthritis, PSQI–Pittsburgh Sleep Quality Index, RCT—Randomized controlled trial, SD—Sleep duration, SE–Sleep Efficiency, SOL–Sleep Onset Latency, SQ–Sleep Quality, SQQ–Sleep Quality Questionnaire, TWT–Total Wake Time

**Table 4:** Information (i.e. aim, study design, primary focus of the study, measure of sleep used, sample, pertinent results relating to sleep) and quality scores for each study using an unvalidated measure of sleep included in the review.

Type of measure of sleep	Study	Aim	Study Design	Primary focus on sleep	Measure of Sleep	Participants	Results pertaining to sleep	Quality Score	Quality Percentage	Quality Decision
HRQoL (unvalidated subjective)	Takahasi et al., 2013	Investigated the effect of various treatments on QoL and mental health of psoriasis patients	Cross-sectional study	No	GHQ-30	199 patients with psoriasis vulgaris	Biologics, other treatments and topical treatments all resulted in significant reductions in sleep disturbance from pre-to-post treatment, with biologics having the greatest effect (P < 0.05)	21/32	65.6	fair
HRQoL/Medical Record (unvalidated subjective)	Sanchez-Carazo et al., 2014	Analyse the clinical profile of patients with moderate-to-severe psoriasis with regards to comorbid conditions and to establish its correlation with QoL	Cross-sectional survey	No	Medical history/SF-36	1022 patients with psoriasis	Moderate-severe psoriasis patients who possessed a diagnosis of a sleep disorder (12.2%) had significantly lower SF-36 scores (P < 0.05)	26/30	86.7	good
HRQoL (unvalidated subjective)	Kim et al., 2013	Examine the relative effects of psoriasis and obesity on Chronic QoL by analysing the physical and psychological burden of the disease that accumulate across the lifespan	Cross-sectional survey	No	Modified DLQI (Chronic QoL) to include questions about social and psychological problems due to psoriasis	114 patients with psoriasis	Sleep problems were significant across the lifetime (p<0.05) for those with higher BMI.	24/30	80	good
HRQoL (unvalidated subjective)	Oostveen et al., 2012	Longitudinal assessment of QoL in juvenile psoriasis	Longitudinal study	No	CDLQI	125 children with psoriasis	Sleep disturbance as measured by CDLQI reduced significantly from initial visit to follow up across all treatments (P < 0.05), with it having the greatest improvement along with itch.	25/30	83.3	good
Pain questionnaire (unvalidated subjective)	Ljosaa et al., 2010	Describe the prevalence of skin pain and discomfort in psoriasis patients, whether skin pain/discomfort differed on demographic and clinical levels and to explore associated symptom characteristics	Cross-sectional study	No	BPI (30)	139 patients with psoriasis	Sleep was the most severely disrupted function (P < 0.05) reported by 74/139 participants.	27/30	90	good
General Health (unvalidated subjective)	Sharma et al., 2001	Evaluate psychiatric morbidity associated with psoriasis and vitiligo	Case-control study	No	GHQ-H	30 patients with psoriasis or vitiligo	Sleep disturbance was the most common complaint, reported by 56.7% of psoriasis patients	23/32	71.9	fair
Pain questionnaire (unvalidated subjective)	Yosipovitch et al., 2000	Assess the prevalence of itch in extensive psoriasis in an outpatient clinic and to assess its clinical pattern	Cross-sectional study	No	Questionnaire based on the McGill Pain Questionnaire	101 patients with psoriasis	69% of patients report that itch results in difficulty falling asleep, with 66% being woken up as a result of itch	25/30	83.3	good
Clinical Interview	Nyunt et al., 2013	Determine the impact of psoriasis on HRQoL, examine the factors associated with HRQoL impairment and determine predictive factors of severe impact of psoriasis on HRQoL	Cross-sectional study	No	Clinical interview	223 patients with psoriasis	Sleep disturbance as reported via clinical interview was significantly associated with severe reductions in DLQI score (P < 0.05)	25/30	83.3	good
Medical records	Tsai et al., 2011	Describe the epidemiology of psoriasis and the prevalence of comorbidities in Taiwanese psoriasis patients	Cross-sectional study	No	Medical Records	51,800 patients with psoriasis	Sleep disorders had a significantly increased prevalence ratio in psoriasis patients (3.89 [2.26, 6.71) (p<0.05.)	23/38	82.14	good
Medical records	Egeberg et al., 2016	To examine the bidirectional impact of psoriasis and sleep apnoea	Cohort study	Yes	Medical Records	66,523 patients with psoriasis	Psoriasis was associated with elevated risk of obstructive sleep apnoea even when adjusting for age, sex, alcohol, comorbidities and socioeconomic status. (mild psoriasis: IRR: 1.30, 95% CI: 1.17–1.44, severe psoriasis: IRR: 1.65, 95% CI:1.23–2.22)	26/30	86.7	good
Medical records	Chiu et al., 2016	Investigate the association between cardiovascular risk and sleep disorders in psoriasis	Cohort study	Yes	Medical Records	99,628 patients with psoriasis	Sleep disorders were significantly associated with increased cardiovascular risk (aHR: 1.25, 95% CI:1.22–1.38) and stroke (aHR: 1.24, 95% CI:1.1–1.33)	23/26	88.5	good
Unvalidated individual question	Duffin et al., 2009	Determine what aspects of psoriasis and psoriatic arthritis are predictive of sleep disturbance using the National Psoriasis Foundation patient surveys	Cross-sectional study	Yes	Individual question ('In a typical month how many days did your disease interfere with your sleeping?')	420 individuals with psoriasis	Psoriatic arthritis and itch were significant predictors of sleep disturbance (P < 0.05).	21/30	70	fair
proxy HRQoL (unvalidated subjective)	Hu et al., 2010	Pilot a WTP instrument and evaluate its feasibility in measuring HRQoL domains within PsA	Cross-sectional study	No	Willingness-to-pay paradigm	59 patients with psoriasis and PsA	The highest median amount of money individuals were willing to pay for a cure was applied to sleep ($10,000)	23/28	82.1	good
proxy HRQoL (unvalidated subjective)	Delfino et al., 2008	Pilot a WTP instrument and evaluate its feasibility in measuring HRQoL domains within Psoriasis and to identify areas of HRQoL most severely affected by psoriasis	Cross-sectional study	No	Willingness-to-pay paradigm	40 patients with psoriasis	Sleep was allocated the lowest median amount of money by patients ($625), but was reported by 22/40 participants as being present.	22/28	78.6	fair
Itch questionnaire (unvalidated subjective)	Amatya et al., 2008	Characterize pruritus and its aggravating and relieving factors and to assess the effect of treatment and the impact of itch on QoL in psoriasis	Cross-sectional study	No	Pruritus questionnaire with one sleep item	80 patients with psoriasis	35% of individuals report itch as interfering with their sleep, and 65% report that good sleep improves itch	23/30	76.7	fair
Unvalidated sleep questionnaire	Gupta & Gupta., 1989	Comparison of the dermatological and psychosocial factors of two psoriasis groups, both of whom report severe itch during wakefulness with and without frequent nocturnal awakenings from sleep	Case-control study	Yes	Sleep questionnaire assessing discomfort during sleep	79 patients with psoriasis (46 with nocturnal awakenings and 33 without)	W group reported greater discomfort due to shedding (P < 0.05); heat intolerance (P < 0.05); cold intolerance (P < 0.05) and jerking of limbs during sleep (P < 0.05), and increased presence of depression than the without awakenings group. No significant differences relating to pruritus.	22/30	73.3	Fair
Unvalidated sleep question	Krueger et al., 2001	Assess patient's views on the impact of psoriasis on their life and emotional wellbeing, along with obtaining their views and satisfaction of current treatments available	Cross-sectional survey	No	Single question asking what activities of daily living are impacted by psoriasis	17,488	Sleep was the second most disrupted activity of daily living, indicated by 20% of 18–34 year olds, 22% of 35–54 year olds and 22% of those >55.	20/28	71.4	Fair

**BMI**—Body Mass Index, **BPI (30)–**Brief Pain Inventory, **(C)DLQI**—(Children’s) Dermatology Life Quality Index, **GHQ-30** –General Health Questionnaire (30) **HRQoL/QoL—**Health related quality of life/Quality of Life, **SF-36** –Short Form 36 Health Survey

**Table 5:** Information (i.e. aim, study design, primary focus of the study, measure of sleep used, sample, pertinent results relating to sleep) and quality scores for the qualitative included in the review.

Type of measure of sleep	Study	Aim	Study Design	Primary focus on sleep	Measure of Sleep	Participants	Results pertaining to sleep	Quality Score	Quality Percentage	Quality Decision
Focus groups and interviews	Globe et al., 2009	Develop a disease model of psoriasis to identify the most important domains to psoriasis patients through physician interviews and patient focus groups	Qualitative study	No	Focus group	31 patients with psoriasis and 5 dermatologists	Patients reported that they experienced difficulty falling asleep, waking up, having non restorative sleep and sleeping less	19/22	86.4	good

### Quality Evaluation

We adapted a standard quality scoring tool [34] for quantitative and qualitative study designs to include information about the quality of the sleep measure used, details of potential confounding variables and the sleep findings reported by each paper. Members of the review team (ALH, SB, CB, AC) tested the quality tool on a sample of four papers. The first author then reviewed all papers, discussing any problems with the fourth and sixth authors with amendments discussed and agreed upon during subsequent meetings until there was full agreement on the domains included in the tool. Subsequently ten randomly selected papers were coded by the third author (SB) independently with these scores compared to those of the first author (ALH). Disagreement in scores was found for one domain which was resolved following discussion with the rest of the team resulting in 100% agreement.

Fifteen domains were used for quantitative papers and seven for qualitative papers (supplementary data). Each quality evaluation domain had specific criteria that had to be fulfilled in order to obtain a score from 0 to 2. A standardised rating scale was used to score each item thus: a score of 2 indicates that criteria had been fulfilled, a score of 1 indicates partial fulfillment, and a score of 0 indicates no fulfillment of the criteria. Quantitative studies, depending on their design could score a maximum score of 40, whereas qualitative studies could score a maximum of 22 points.

Each paper's total quality score was then standardised as a percentage, obtained by dividing the study’s score by the total points available. Quality appraisal thresholds determined by the team were then applied to each study based upon its percentage score (good: ≥80%; fair: ≥61–79%; and poor: ≤60%).

## Results

**Fig 1:**
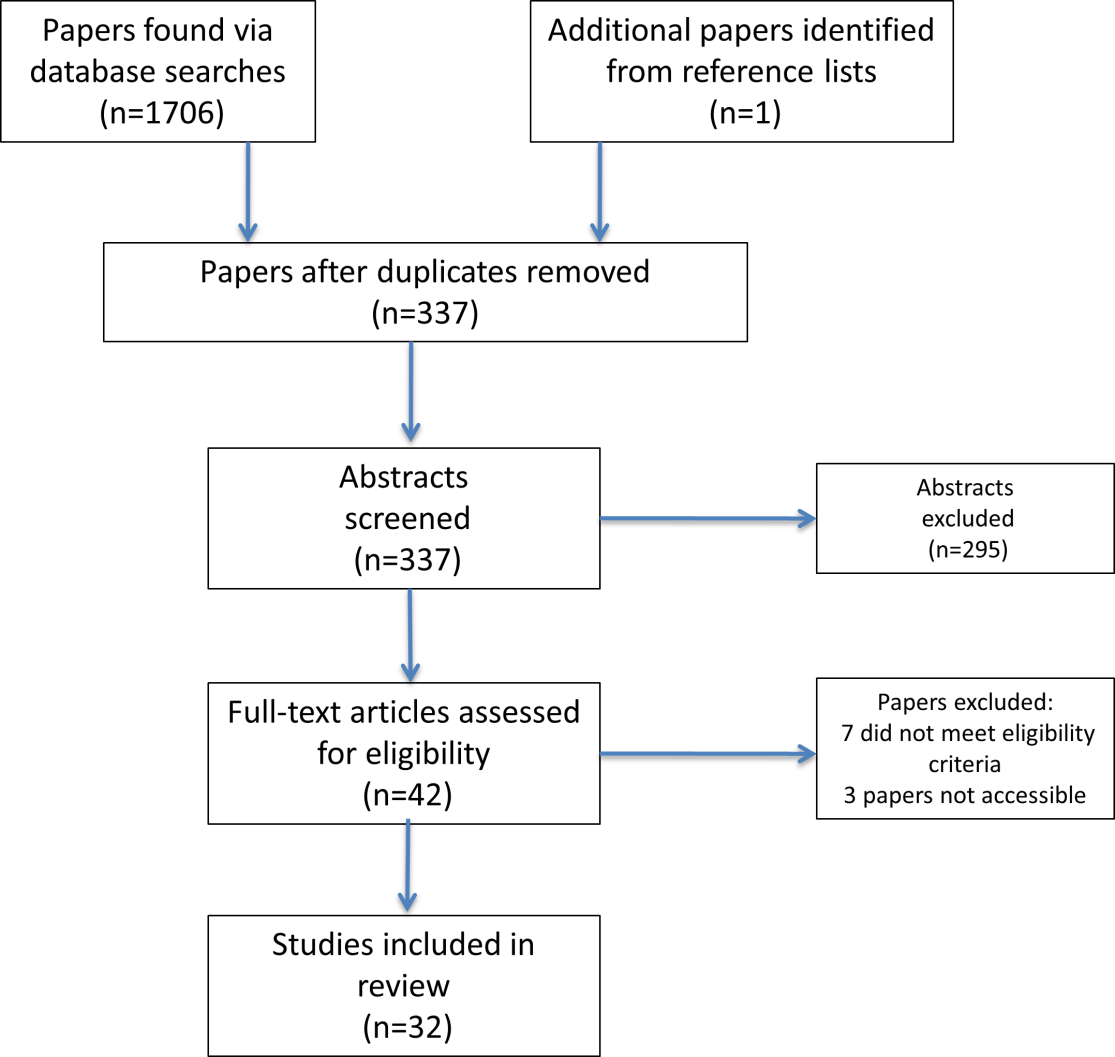
PRISMA Flow diagram outlining the systematic review process.

We identified 337 original papers. Abstracts were screened for eligibility and 42 full papers were obtained and scrutinised (Fig 1). In total, 32 papers were included in the final review. Thirty-one studies employed quantitative designs, and one study used a qualitative framework. We structure our review findings under the following category headings: study quality; sleep methodology and prevalence of sleep disruption; and factors associated with sleep disturbance.

### Study Quality

Fewer than half of all studies (15/32; 46.9%) were rated as good quality (i.e. scoring ≥ 80%), over fair (15/32;46.9%) (scoring ≥ 61–79%), and the remainder (2/32; 6.25%) as poor (scoring ≤ 60%) (Tables 3–5). Overall quality scores were fair (mean % = 76.9), with a number of methodological limitations identified across studies: 27 studies failed to perform sample size calculations, 17 had inappropriately small or large sample sizes, 7 studies did not provide demographic data, 2 studies failed to report any data on central tendency or variability, and 5 studies reported this data for only one time point and 14 studies failed to control for confounding variables (S1 Table). Of the 14 studies using valid measures of sleep, the majority (9) scored in fair (scoring ≥ 61–79%), and poor (2) categories (scoring ≤ 60%) and only 3 were rated as good (scoring ≥80%).

### Measurement and prevalence of sleep disturbance

Studies used a range of sleep measures but only in 14 of 31 (45.2%) had these been validated. Five studies used polysomnography (PSG), and focused on OSA [26,27,35,36] or itch [37], with one of these also using the Functional Outcomes of Sleep Questionnaire [26]. Four used the Pittsburgh Sleep Quality Index (PSQI) [23,24,38,39], one of which was conducted on a PsA sample [39] and one study used a condensed version of the PSQI [40]. One study used the Insomnia Severity Index (ISI) [23] while a further four studies used other validated self-report measures specifically, the General Sleep Disturbance Scale [41] and the Medical Outcomes Study Sleep Scale (MOS-SS) [28,42,43]. One study was qualitative, evaluating the impact of pruritus using interviews with physicians alongside patient focus groups [44].

The remaining 17 (54.8%) quantitative studies used non-validated sleep measures. Six studies used broad measures of health and functioning, which incorporated sleep items, such as measures health-related quality of life (HRQoL) (General Health Questionnaire, modified Dermatology Life Quality Index, Children’s Dermatology Life Quality Index, General Health Questionnaire Hindi) or other general health-related questionnaires (Brief Pain Inventory, McGill Pain Questionnaire) [45–50]. Four studies used non-validated questionnaires [25,51–53] three of which contained a single item pertaining to sleep disturbance [25,52,53] (e.g "in a typical month, how many days did your disease interfere with sleeping?”) [25]. Four studies solely used medical records [54,55,56,57], and another used a clinical diagnostic interview with a sleep disorder diagnosis [58]. In two validation studies HRQoL was assessed using a willingness-to-pay paradigm, where participants were asked to indicate how much money they would allocate to relieve themselves of a particular symptom [59,60]. Interestingly, in one of the studies using a PsA sample, sleep was allocated the highest median amount of money ($10,000) [60], whereas in the other study, with a psoriasis sample, sleep was the lowest ranked issue ($625) [59].

Of all studies reviewed, 11 (34.4%) had an *a priori* aim to assess sleep, however, only three (9.4%) had the primary objective of assessing sleep quality and quantifying sleep disturbance in psoriasis [23,24,37]. The other studies were not *primarily* focused on general sleep in psoriasis. Two studies investigated associations between PsA and sleep [25,39] while four studies assessed the link between OSA and psoriasis [26,27,35,36]. The remaining two studies measured the influence of itch on psoriasis [37,51], one of which used a mixed dermatological sample [37] with just 1 of 15 participants (6.67%) having psoriasis. The former [51] used a questionnaire and the latter [37] used PSG to assess sleep. One study aimed to assess the links between sleep disorders in psoriasis and cardiovascular risk [56].

Thirty (93.7%) of 32 studies, including one qualitative study, observed sleep disturbance. However, in studies where sleep was measured prospectively, values varied from 0.05% to 87.5% (Table 6). Two papers reported no sleep problems relative to healthy controls [24,59]. In one of these studies, whilst sleep disturbance was reported by the majority (55%) of participants, relative to other domains of life, sleep disturbance was the least bothersome [59].

**Table 6:** Rates of sleep disturbance found in studies where sleep was measured or quantified within the sample. Studies where the whole sample had sleep disturbance or where the number of those experiencing sleep disturbance are not included in this table.

	Rates of sleep disruption (%)	Measures used to assess sleep
**Karaca et al.**	18/32 (Dx with OSA) (56.3)	Polysomnography
**Papadavid et al.**	19/35 (Dx with OSA) (54.3)	Polysomnography
**Buslau & Bentomane**	9/25 (Dx with OSA) (36)	Polysomnography
**Gezer et al.**	35/41 (85.4)	PSQI
**Shutty et al.**	81.80%	PSQI
**Ljosaa et al. 2012**	63%	GSDS
**Sharma et al.**	17/30 (56.7)	GHQ-H
**Ljosaa et al. 2010**	74/139 (53.2)	BPI (30)
**Hu et al.**	60/100 (60)	Willingness-to-pay (proxy HRQoL measure)
**Delfino et al.**	22/40 (55)	Willingness-to-pay (proxy HRQoL measure)
**Duffin et al.**	208/420 (49.5)	Individual question ('In a typical month how many days did your disease interfere with your sleeping?')
**Nyunt et al**	91/223 (40.9)	Clinical interview
**Sanchez-Carazo et al.**	125/1022 (12.2)	Medical History
**Chiu et al.**	2,223/99,628 (2.23)	Sleep disorder diagnosis
**Tsai et al.**	28/51,800 (0.05)	Sleep disorder diagnosis

BPI–Brief Pain Inventory, Dx–Diagnosed, GHQ–General Health Questionnaire,

GSDS–General Sleep Disturbance Scale, HRQoL–Health Related Quality of Life,

OSA–Obstructive Sleep Apnea, PSG–Polysomnography, PSQI—Pittsburgh Sleep Quality Index

In contrast, another study reported sleep as being the second-most disrupted domain as a consequence of psoriasis, across young, middle aged and older-aged respondents; indicated by 20%, 22% and 22% respectively [53]. Sleep quality was worse in psoriasis patients (8.8±4.4 vs. 6.3±4.4) [23] and PsA patients (9.70±3.9 vs. 4.05±1.85) [39] relative to healthy controls in two out of three studies using PSQI with the third paper showing no difference between psoriasis patients and controls (5.56 vs. 5.13) [46]. The fourth paper using the PSQI showed that on two components of the measure scores were significantly worse in psoriasis subjects than healthy controls (subjective sleep quality; 1.48±0.17 vs. 1.02±0.13 and habitual sleep efficiency; 0.79±0.19 vs. 0.32±0.14) [38]. Nevertheless, they did not report data for one component nor did they provide global PSQI scores for both groups [38]. Furthermore, one of these studies showed significantly increased ISI scores in psoriasis patients (11.0±7.0) relative to healthy controls (6.3±6.0) [23], with psoriasis patients’ mean score at the threshold for clinical insomnia [61].

Four studies found high rates of OSA in psoriasis patients ranging from 36% to as high as 56.3% [27,35,36]. Moreover in a large epidemiological study, psoriasis patients had a significantly increased prevalence of sleep disorder diagnosis than non-psoriasis counterparts [57] and in another, psoriasis patients with a sleep disorder had elevated cardiovascular risk relative to those without a sleep disorder [56].

### Factors associated with sleep disturbance

Sixteen of the 32 reviewed studies reported factors associated with sleep disturbance. Itch, depression, PsA and pain were the most commonly reported factors associated with sleep disturbance however others were reported across studies. Itch was reported to occur more frequently at night [50] and in six studies it was associated with disrupted sleep [25,37,42,44,50,52]. Itch predicted lower scores on the MOS-SS, indicating worse sleep [42]; increased scratching was associated with more awakenings during stages 1 and 2 of sleep [37]; and itch was reported by subjects as causing difficulty initiating and maintaining sleep as well as performing daily activities [44]. Additionally, in one study 65% of participants reported that sleep ameliorated itch [52].

The association between itch and sleep was documented further in one study finding the association between depressive symptoms and itch severity was partially mediated by sleep quality [40]. In another study daytime sleepiness was associated with depression [28]. Contrasting findings were indicated by one study in which itch levels were reported as equivalent across those with and without sleep disruption, whereas depression was significantly higher in those who experienced sleep disruption relative to those who did not [51]. This suggests that depression may have played a greater role in sleep disruption than itch. This is supported by another study showing that when controlling for depression variables, psoriasis patients are no more likely to experience poor sleep or have greater insomnia symptoms than controls [23], and another study showing that while PSQI scores were higher in psoriasis subjects, there were no differences in psychiatric symptoms relative to controls [38].

Pain appears to contribute to poorer sleep, and in one study over 85% of those reporting increased pain specified sleep as the most disrupted function [47]. Another study showed that sleep disturbance mediated the relationship between pain and reduced HRQoL [41]. Four studies showed associations between PsA and sleep [25,28,39,60]. PsA and generalized pain was associated with reduced PSQI scores relative to healthy controls [39], increased want for improved sleep [59] and predicted sleep disturbance [25]. Additionally sleep disturbance was associated with anxiety, enthesitis, levels of C-reactive protein and erythrocyte sedimentation rate [39].

Sleep disturbance was consistently associated with reduced QOL [28,47,54,58]. Moreover, in four treatment studies using biologics, improvements were observed in sleep quality, disturbance, daytime sleepiness and adequacy [26,41,43,46] suggesting that improvements in psoriasis disease processes may lead to concomitant improvements in sleep. Furthermore in a longitudinal cohort study sleep showed the greatest improvement from baseline to follow-up across varied psoriasis therapies (topicals and systemic) relative to other items on the Children’s Dermatology Life Quality Index [48].

## Discussion

### Key findings from the review

This systematic review examined the evidence for sleep disturbance in psoriasis and the measures and methodology used to assess sleep. The majority of studies showed sleep problems were associated with itch, low mood and pain. Itch appears to be associated with increased sleep fragmentation, disturbance and reduced quality of sleep. These findings are consistent with research in other pruritic conditions [62–64]. Low mood and pain were also implicated in poor sleep as previously shown in both healthy individuals and other illness populations [65, 66]. It is likely that mood, pain and itch interact disturb sleep; although the nature of this interaction requires examination within the context of purposively-designed studies.

Prevalence rates varied substantially and while studies were of fair-to-good quality, many demonstrated a number of methodological and statistical shortcomings. In particular, the lack of validated measures of sleep, coupled with limited theoretical work-up may have accounted for the variation in rates of sleep disturbance across studies. Nevertheless, prevalence clustered around the 50–60% mark; higher than current estimations of sleep pathology in the general population [67]. Additionally, rates of OSA appear to be higher in psoriasis (36–56.3%) relative to the general population (3–7%)[68]. Although the reasons for this are unclear; OSA is multifaceted, involving both inflammatory pathways and lifestyle factors including high BMI and physical inactivity [69]. OSA can have a significant impact on health and contribute to cardiovascular disease risk [70], which is already elevated in psoriasis [4]. Further research should identify mechanisms underpinning the link between OSA and psoriasis.

Lack of detailed assessments and incomplete data reporting have impeded further conclusions and provide a somewhat superficial picture of the true problem. For instance, studies using the PSQI often failed to report full data for the measure (sleep quality, sleep latency, sleep duration, habitual sleep efficiency, sleep disturbance, use of sleeping medication and daytime dysfunction). Providing these data would facilitate a more thorough and itemised understanding of sleep quality.

Moreover, despite the ‘gold standard’ PSG being used in five studies, it provides limited information about the range of factors that may impact upon sleep [71, 72]. Comprehensive assessment of sleep involves the integration of objective and subjective measures, profiling of sleep-wake patterning over time, and the assessment of factors that may contribute to night-to-night variation in sleep quantity and quality [71,72]. We found no studies in this review that conformed to these standards and neither actigraphy nor sleep diaries have been applied in psoriasis populations. These data would support a more nuanced understanding of sleep disruption in psoriasis and help to design appropriate interventions [71,72].

### Limitations

This review only included studies published in English and those that have undergone peer review. Therefore, it is possible that some relevant findings may have been missed. Further, due to the subjective nature of the quality scoring, there is greater opportunity for personal bias to influence scores. However we attempted to minimize this risk by the authors scoring papers independently and there was close to 100% agreement in paper scores following independent scoring.

### Concluding remarks: Recommendations for future research

There is a need to systematically and consistently examine sleep in psoriasis populations, employing comprehensive and validated measures of sleep in specifically designed studies. Accurate prevalence rates of sleep disturbance must first be established; from here prospective studies can explore the relationship between sleep and psoriasis, focusing on potential precipitating and perpetuating factors that may drive and maintain poor sleep, (candidate targets: itch, mood, pain and OSA). Work should also explore sleep-wake variability in this population and assess any concomitant consequences on daytime functioning.

Obtaining a comprehensive understanding of sleep in psoriasis is of importance due to the implications of sleep disruption for health. Sleep disturbance may result in disruptions to immune and sympathetic nervous system functioning possibly leading to the maintenance and/or exacerbation of psoriasis, conferring risk for adverse psychological and medical outcomes. This has been suggested in a recent study which found that the presence of a sleep disorder alongside psoriasis confers increased risk for CVD morbidity [56].

The potential role of inflammation in the relationship between poor sleep and psoriasis has also been suggested. Four studies included in this review [26,41,43,46] showed improvements but not a mitigation of sleep problems following the administration of biologic medication. This suggests that reductions in systemic inflammation may confer sleep improvement possibly through a reduction in itch and associated skin discomfort. Further research is required to examine mediating pathways and treatment mechanisms in greater detail.

## Supporting Information

S1 Table Domain scores for all studies included in the review. (DOCX)

S2 Table PRISMA Checklist. (DOC)
