# Supplementary figures and images for: Measurement, Classification and Evaluation of Sleep Disturbance in Psoriasis: A Systematic Review
Source: PLoS One. 2016 Jun 21;11(6):e0157843. doi: 10.1371/journal.pone.0157843 (PMC4915697; doi:10.1371/journal.pone.0157843)

Appendix 1: Domain Scores for all studies included in review (a) = quantitative, (b) = qualitative

(a)


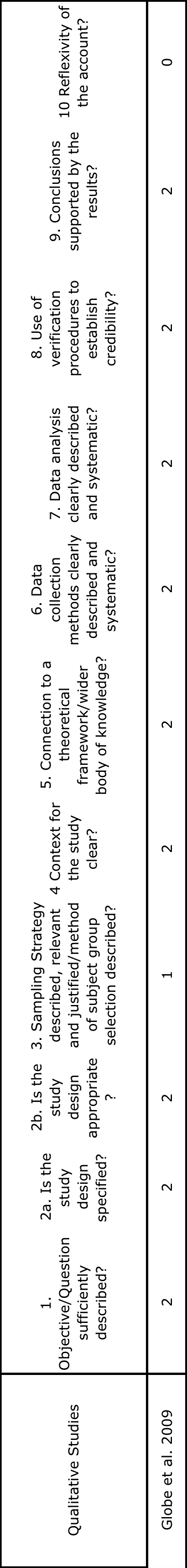


(b)

Supplement: S1 Table — (DOCX) [file pone.0157843.s001.docx]
